# Crystal structure and Hirshfeld surface analysis of a chalcone derivative: (*E*)-3-(4-fluoro­phen­yl)-1-(4-nitro­phen­yl)prop-2-en-1-one

**DOI:** 10.1107/S2056989018017450

**Published:** 2019-01-01

**Authors:** Qin Ai Wong, Tze Shyang Chia, Huey Chong Kwong, C. S. Chidan Kumar, Ching Kheng Quah, Md. Azharul Arafath

**Affiliations:** a X-ray Crystallography Unit, School of Physics, Universiti Sains Malaysia, 11800 USM, Penang, Malaysia; b School of Chemical Sciences, Universiti Sains Malaysia, 11800 USM, Penang, Malaysia; c Department of Engineering Chemistry, Vidya Vikas Institute of Engineering and Technology, Visvesvaraya Technological University, Alanahalli, Mysuru 570 028, India; d Department of Chemistry, Shahjalal University of Science and Technology, Sylhet, 3114, Bangladesh

**Keywords:** crystal structure, chalcone, hydrogen bond, Hirshfeld surface analysis

## Abstract

The mol­ecular structure of the title chalcone derivative, C_15_H_10_FNO_3_, is nearly planar and the mol­ecule adopts a *trans* configuration with respect to the C=C double bond. The nitro group is nearly coplanar with the attached benzene ring, which is nearly parallel to the second benzene ring. In the crystal, mol­ecules are connected by pairs of weak inter­molecular C—H⋯O hydrogen bonds into inversion dimers. The dimers are further linked by another C—H⋯O hydrogen bond and a C—H⋯F hydrogen bond into sheets parallel to (104). π–π inter­actions occur between the sheets, with a centroid–centroid distance of 3.8860 (11) Å. Hirshfeld surface analysis was used to investigate and qu­antify the inter­molecular inter­actions.

## Abstract 2

The mol­ecular structure of the title chalcone derivative is nearly planar and the mol­ecule adopts a *trans*-configuration with respect to the conjugated C=C double bond. In the crystal, the mol­ecules are connected by weak inter­molecular C—H⋯O and C—H⋯F hydrogen bonds into sheets parallel to (104). Weak inter­molecular π–π inter­actions also occur.

## Chemical context   

Non-linear optics (NLO) is the study of inter­actions between intense light and matter, in which the dielectric polarization responds non-linearly to the electric field of the light. This non-linearity leads to frequency-mixing processes (second-, third- and high-harmonic generations), the optical Kerr effect *etc* (Boulanger & Zyss, 2006 ▸). Chalcone is one of the NLO materials and is known for its high NLO coefficients and good crystallizability (Prabhu *et al.*, 2013 ▸). Donor–acceptor substituted chalcone derivatives consist of two substituted phenyl rings covalently bonded to the ends of a α,β-unsaturated propenone bridge (C=C—C=O), which provides the necessary configuration for intra­molecular charge transfer to show NLO properties (Fun *et al.*, 2011 ▸). However, organic chalcone derivatives with a low melting point are at a disadvantage for applications as optical instruments. In a contin­uation of our ongoing studies on non-linear optical properties of various chalcone derivatives (Chandra Shekhara Shetty *et al.*, 2017 ▸; Ekbote *et al.*, 2017 ▸; Kwong *et al.*, 2018 ▸), we report herein the synthesis, structure determination and Hirshfeld surface analysis of the title compound.
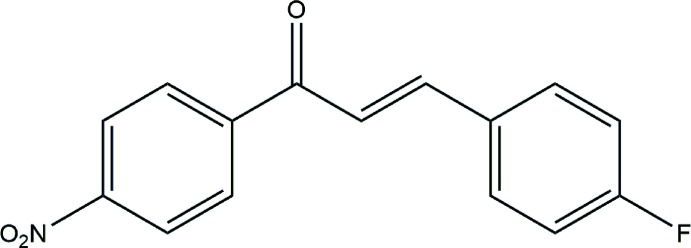


## Structural commentary   

**Figure 1:**
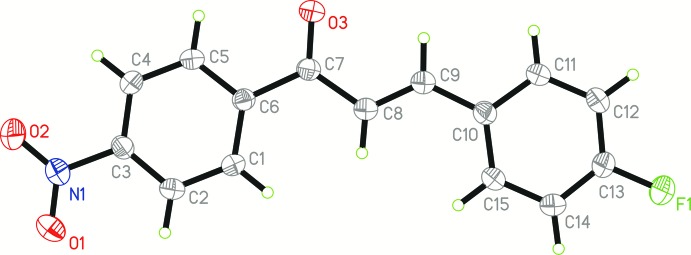
The mol­ecular structure of the title compound with atom labels and 30% probability displacement ellipsoids.

The asymmetric unit of the title chalcone derivative consists of a unique mol­ecule, containing two *para*-substituted phenyl rings and an enone connecting bridge (Fig. 1 ▸). The mol­ecule adopts a *trans* configuration with respect to the C8=C9 olefinic double bond, as indicated by the C7—C8—C9—C10 torsion angle of −179.96 (15)°. The C7=O3 carbonyl group adopts an *s*-*cis* configuration with respect to the C8=C9 double bond as indicated by O3—C7—C8—C9 torsion angle of −0.8 (3)°. The mol­ecule (excluding H atoms) is nearly planar with a maximum deviation of 0.103 (2) Å at atom O1 of the terminal nitro group. The nitro group is nearly coplanar with the attached C1–C6 benzene ring as indicated by the small dihedral angle of 7.9 (2)°. The C1–C6 and C10–C15 benzene rings make a small dihedral angle of 4.27 (8)° with each other.

## Supra­molecular features   

**Figure 2:**
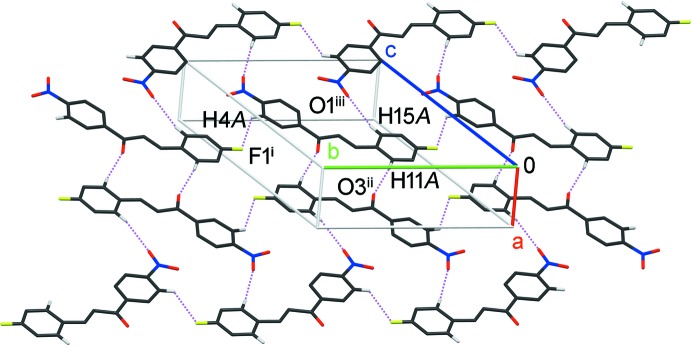
A partial packing diagram of the title compound, showing a two-dimensional sheet formed by C—H⋯O and C—H⋯F hydrogen bonds (dotted lines). H atoms not involved in hydrogen bonding are omitted for clarity. [Symmetry codes: (i) *x*, *y* + 1, *z*; (ii) −*x* + 2, −*y* + 1, −*z* + 1; (iii) −*x*, *y* − 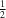, −*z* + 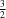.]

**Figure 3:**
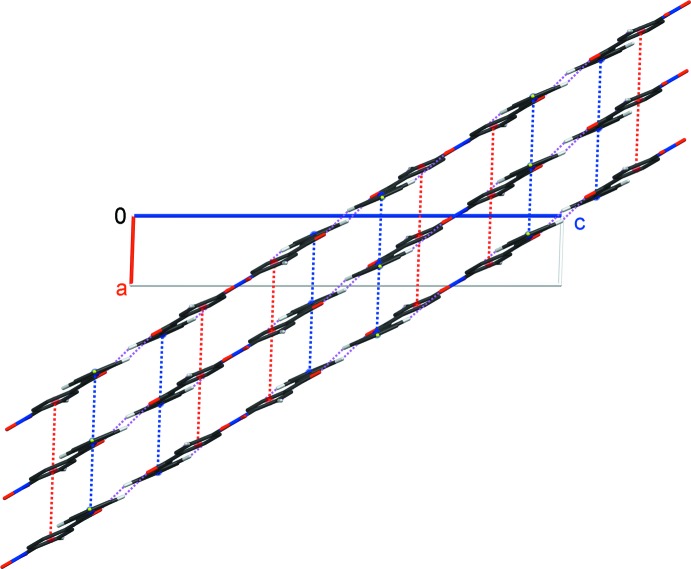
A partial packing diagram of the title compound, showing three separated sheets parallel to (104). The inter­molecular π–π inter­actions between adjacent sheets are represented as red and blue dashed lines, involving *Cg*1⋯*Cg*1 and *Cg*2⋯*Cg*2, respectively. *Cg*1 and *Cg*2 are the centroids of the C1–C6 and C10–C15 benzene rings, respectively.

In the crystal, mol­ecules are connected by pairs of weak C—H⋯O hydrogen bonds (C11—H11*A*⋯O3^ii^; symmetry code as in Table 1 ▸) into inversion dimers with an 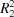
(14) ring motif. These dimers are further linked by C—H⋯O and C—H⋯F hydrogen bonds (C15—H15*A*⋯O1^iii^ and C4—H4*A*⋯F1^i^; Table 1 ▸) into two-dimensional sheets parallel to (104) (Fig. 2 ▸). Weak π–π inter­actions occur between the sheets [*Cg*1⋯*Cg*1^iv,v^ and *Cg*2⋯*Cg*2^iv,v^ = 3.8860 (11) Å, where *Cg*1 and *Cg*2 are the centroids of C1–C6 and C10–C15 benzene rings, respectively; symmetry codes: (iv) *x* − 1, *y*, *z*; (v) *x* + 1, *y*, *z*] (Fig. 3 ▸).

## Hirshfeld surface analysis   

**Figure 4:**
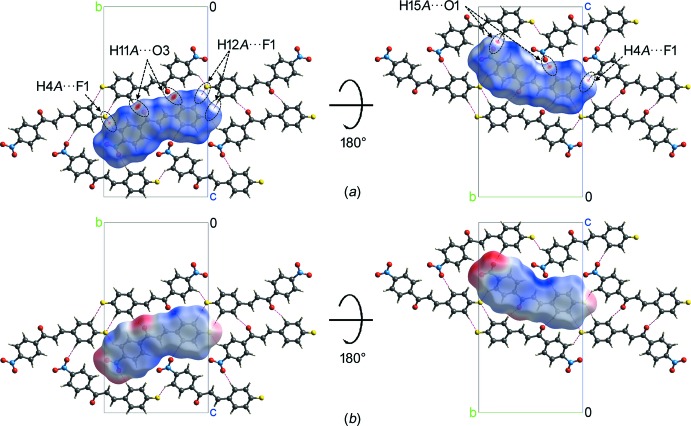
The Hirshfeld surfaces mapped with (*a*) *d*_norm_ and (*b*) electrostatic potential for the central mol­ecule of the title compound surrounded by six neighbouring mol­ecules.

**Figure 5:**
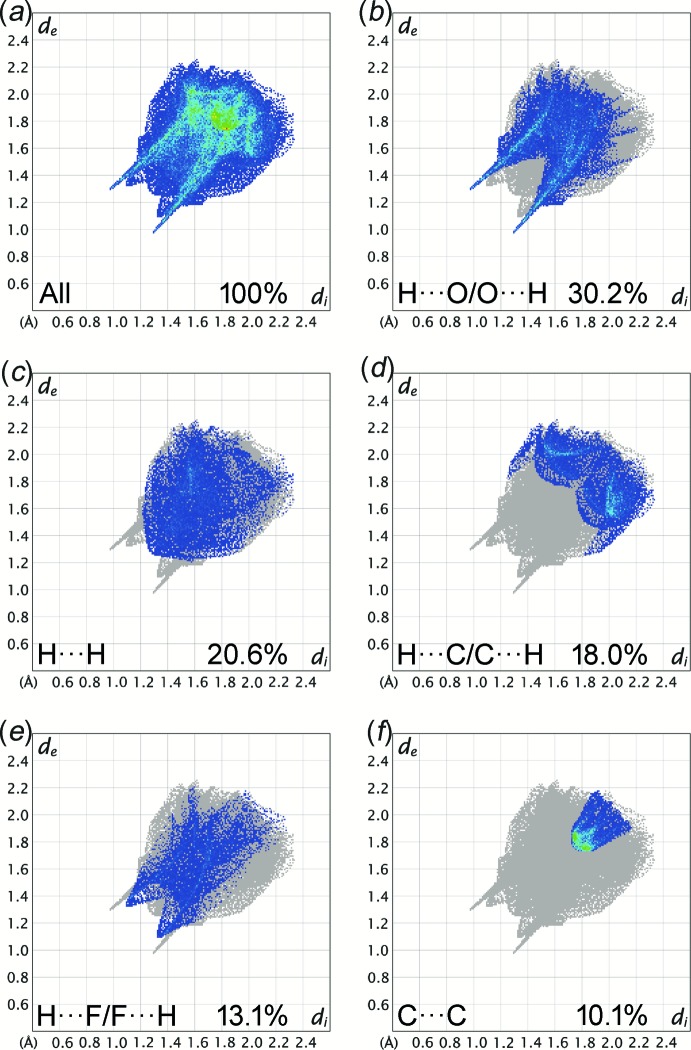
The two-dimensional fingerprint plots of the title compound for different inter­molecular contacts and their percentage contributions to the Hirshfeld surface. *d*_i_ and *d*_e_ are the distances from the Hirshfeld surface to the nearest atom inter­ior and exterior, respectively, to the surface.

**Figure 6:**
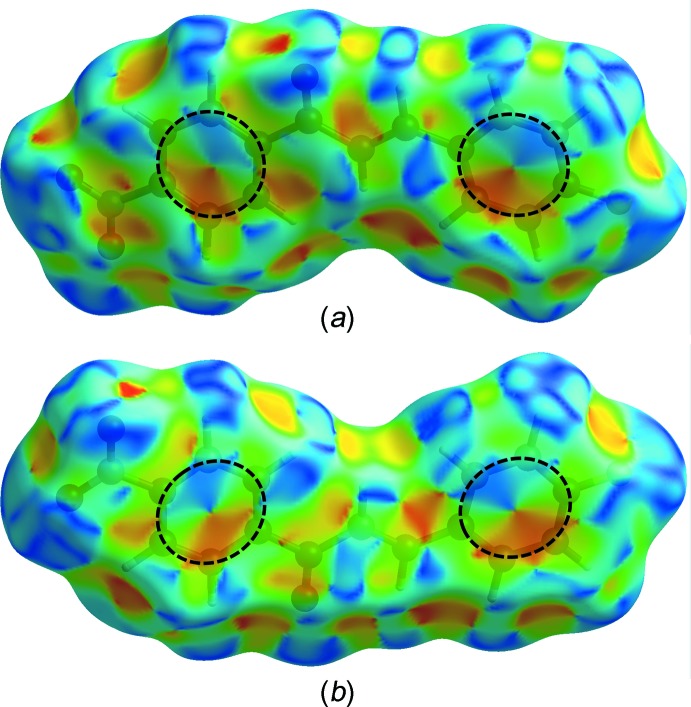
(*a*) Front and (*b*) rear views of the Hirshfeld surface mapped over shape-index for the title compound. The dashed-line circles highlight unique patterns of red and blue ‘triangles’.

**Figure 7:**
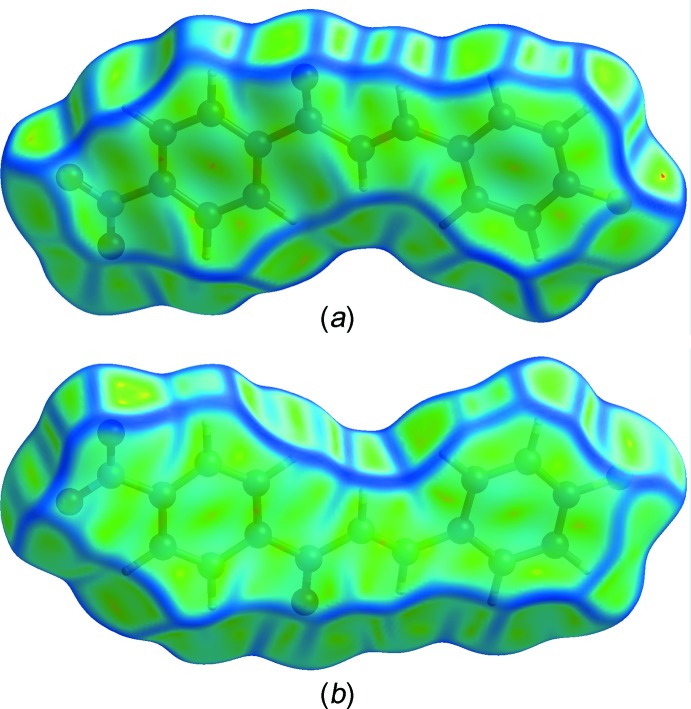
(*a*) Front and (*b*) rear views of the Hirshfeld surface mapped over curvedness.

The Hirsheld surfaces mapped with normalized contact distance *d*_norm_ and electrostatic potentials, and the two-dimensional fingerprint plot were generated using *CrystalExplorer* (Version 17.5; Spackman & Jayatilaka, 2009 ▸; Spackman & McKinnon, 2002 ▸; Spackman *et al.*, 2008 ▸; Turner *et al.*, 2017 ▸). The darkest red spots on the Hirshfeld surface mapped with *d*_norm_ [Fig. 4 ▸(*a*)] correspond to the C11—H11*A*⋯O3 hydrogen bond. The C4—H4*A*⋯F1 and C15—H15*A*⋯O1 hydrogen bonds are indicated as two pairs of lighter red spots on the *d*_norm_ surface. The H12*A*⋯F1 contact, with its H⋯F distance shorter than the sum of van der Waals radii by 0.01 Å, appears as two tiny red spots on the *d*_norm_ surface. The donor and acceptor of a hydrogen bond with positive and negative electrostatic potentials, respectively, are represented as blue and red regions on the Hirshfeld surface mapped with electrostatic potential [Fig. 4 ▸(*b*)]. The electrostatic potential of the F atom is less negative as compared to the O atoms of nitro and carbonyl groups, as indicated by the lighter red region. The H⋯O/O⋯H contacts are the most populated contacts and contribute 30.2% of the total inter­molecular contacts, followed by H⋯H (20.6%), H⋯C/C⋯H (18.0%), H⋯F/F⋯H (13.1%) and C⋯C (10.1%) contacts (Fig. 5 ▸). The shortest H⋯O/O⋯H and H⋯F/F⋯H contacts are represented as the tips of the pseudo-mirrored sharp spikes and blunt peaks at *d*_e_ + *d*_i_ ≃ 2.3 and 2.4 Å, respectively, which correspond to the C11—H11*A*⋯O3 and C4—H4*A*⋯F1 hydrogen bonds. The characteristic ‘wings’ are missing in the fingerprint plot of H⋯C/C⋯H contacts, indicating the absence of any significant C—H⋯π inter­actions in the crystal. The C⋯C contacts, including the inter­molecular π–π inter­actions, appear as a unique ‘triangle’ focused at *d*_e_ ≃ *d*_i_ ≃ 1.8 Å. The presence of significant π–π inter­actions is supported by the unique pattern of red and blue ‘triangles’ on the shape-index surface (Fig. 6 ▸), and the flat regions on the curvedness surface (Fig. 7 ▸) of the benzene rings.

## Database survey   

The bond lengths and bond angles of the title compound are comparable with those in two similar structures, *viz.*, (*E*)-1-(4-nitro­phen­yl)-3-phenyl­prop-2-en-1-one (refcode BUDXOO; Jing, 2009*a* ▸) and (*E*)-3-(4-fluoro­phen­yl)-1-phenyl­prop-2-en-1-one (refcode BUDYOP; Jing, 2009*b* ▸) found in the Cambridge Structural Database (Version 5.39; Groom *et al.*, 2016 ▸). The mol­ecular conformations of these two structures are nearly planar, with small dihedral angles of 5.00 (6) and 10.60 (11)°, respectively, between the phenyl rings.

## Synthesis and crystallization   

4-Nitro­aceto­phenone (1.65 g, 0.01 mol) and 4-fluoro­benzaldehyde (1.24 g, 0.01 mol) were dissolved in methanol (20 ml). A catalytic amount of NaOH was added to the solution dropwise with vigorous stirring. The reaction mixture was stirred for about 6 h at room temperature. The progress of the reaction was monitored by TLC. The formed crude product was filtered, washed repeatedly with distilled water and recrystallized from ethanol to obtain the title chalcone derivative. Yellowish single-crystals suitable for X-ray diffraction were obtained from an acetone solution by slow evaporation at room temperature.

## Refinement   

Crystal data, data collection and structure refinement details are summarized in Table 2 ▸. All H atoms were positioned geometrically (C—H = 0.93 Å) and refined using a riding model with *U*_iso_(H) = 1.2*U*_eq_(C).

## Supplementary Material

Crystal structure: contains datablock(s) I. DOI: 10.1107/S2056989018017450/is5506sup1.cif

Structure factors: contains datablock(s) I. DOI: 10.1107/S2056989018017450/is5506Isup2.hkl

Supporting information file. DOI: 10.1107/S2056989018017450/is5506Isup3.cml

CCDC reference: 1036743

Additional supporting information:  crystallographic information; 3D view; checkCIF report

## Figures and Tables

**Table 1:** Hydrogen-bond geometry (Å, °)

*D*—H⋯*A*	*D*—H	H⋯*A*	*D*⋯*A*	*D*—H⋯*A*
C4—H4*A*⋯F1^i^	0.93	2.53	3.183 (2)	128
C11—H11*A*⋯O3^ii^	0.93	2.43	3.329 (2)	161
C15—H15*A*⋯O1^iii^	0.93	2.58	3.489 (2)	166

Symmetry codes: (i) 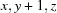; (ii) 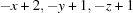; (iii) 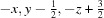.

**Table 2:** Experimental details

Crystal data	
Chemical formula	C_15_H_10_FNO_3_
*M*_r_	271.24
Crystal system, space group	Monoclinic, *P*2_1_/*c*
Temperature (K)	296
*a*, *b*, *c* (Å)	3.8860 (5), 13.2324 (16), 24.199 (3)
β (°)	91.963 (2)
*V* (Å^3^)	1243.6 (3)
*Z*	4
Radiation type	Mo *K*α
μ (mm^−1^)	0.11
Crystal size (mm)	0.49 × 0.35 × 0.31

Data collection	
Diffractometer	Bruker SMART APEXII DUO CCD area-detector
Absorption correction	Multi-scan (*SADABS*; Bruker, 2009 ▸)
*T*_min_, *T*_max_	0.794, 0.926
No. of measured, independent and observed [*I* > 2σ(*I*)] reflections	10823, 2418, 1922
*R*_int_	0.026
(sin θ/λ)_max_ (Å^−1^)	0.617

Refinement	
*R*[*F* ^2^ > 2σ(*F* ^2^)], *wR*(*F* ^2^), *S*	0.043, 0.138, 1.04
No. of reflections	2418
No. of parameters	181
H-atom treatment	H-atom parameters constrained
Δρ_max_, Δρ_min_ (e Å^−3^)	0.21, −0.17

Computer programs: *APEX2* and *SAINT* (Bruker, 2009 ▸), *SHELXS2013* and *SHELXTL* (Sheldrick, 2008 ▸), *SHELXL2014* (Sheldrick, 2015 ▸), *Mercury* (Macrae *et al.*, 2008 ▸), *PLATON* (Spek, 2009 ▸) and *publCIF* (Westrip, 2010 ▸).

## supplementary crystallographic information

### Crystal data

**Table:**

C_15_H_10_FNO_3_	*F*(000) = 560
*M**_r_* = 271.24	*D*_x_ = 1.449 Mg m^−^^3^
Monoclinic, *P*2_1_/*c*	Mo *K*α radiation, λ = 0.71073 Å
*a* = 3.8860 (5) Å	Cell parameters from 4607 reflections
*b* = 13.2324 (16) Å	θ = 2.3–30.4°
*c* = 24.199 (3) Å	µ = 0.11 mm^−^^1^
β = 91.963 (2)°	*T* = 296 K
*V* = 1243.6 (3) Å^3^	Block, yellow
*Z* = 4	0.49 × 0.35 × 0.31 mm

### Data collection

**Table:**

Bruker SMART APEXII DUO CCD area-detector diffractometer	2418 independent reflections
Radiation source: fine-focus sealed tube	1922 reflections with *I* > 2σ(*I*)
Graphite monochromator	*R*_int_ = 0.026
φ and ω scans	θ_max_ = 26.0°, θ_min_ = 1.8°
Absorption correction: multi-scan (*SADABS*; Bruker, 2009)	*h* = −4→4
*T*_min_ = 0.794, *T*_max_ = 0.926	*k* = −16→15
10823 measured reflections	*l* = −29→29

### Refinement

**Table:**

Refinement on *F*^2^	0 restraints
Least-squares matrix: full	Hydrogen site location: inferred from neighbouring sites
*R*[*F*^2^ > 2σ(*F*^2^)] = 0.043	H-atom parameters constrained
*wR*(*F*^2^) = 0.138	*w* = 1/[σ^2^(*F*_o_^2^) + (0.072*P*)^2^ + 0.3042*P*] where *P* = (*F*_o_^2^ + 2*F*_c_^2^)/3
*S* = 1.04	(Δ/σ)_max_ = 0.001
2418 reflections	Δρ_max_ = 0.21 e Å^−^^3^
181 parameters	Δρ_min_ = −0.17 e Å^−^^3^

### Special details

**Table:**

Geometry. All esds (except the esd in the dihedral angle between two l.s. planes) are estimated using the full covariance matrix. The cell esds are taken into account individually in the estimation of esds in distances, angles and torsion angles; correlations between esds in cell parameters are only used when they are defined by crystal symmetry. An approximate (isotropic) treatment of cell esds is used for estimating esds involving l.s. planes.

### Fractional atomic coordinates and isotropic or equivalent isotropic displacement parameters (Å^2^)

**Table:**

	*x*	*y*	*z*	*U*_iso_*/*U*_eq_	
F1	0.7362 (4)	−0.01394 (9)	0.57883 (5)	0.0899 (5)	
O1	−0.1032 (4)	0.85915 (12)	0.77829 (6)	0.0784 (5)	
O2	0.0898 (6)	0.97163 (12)	0.72386 (8)	0.1026 (7)	
O3	0.7296 (4)	0.61266 (9)	0.55381 (5)	0.0665 (4)	
N1	0.0476 (4)	0.88363 (12)	0.73723 (6)	0.0578 (4)	
C1	0.3037 (4)	0.63109 (12)	0.68456 (6)	0.0460 (4)	
H1A	0.2995	0.5638	0.6955	0.055*	
C2	0.1758 (4)	0.70499 (13)	0.71878 (6)	0.0479 (4)	
H2A	0.0857	0.6880	0.7527	0.057*	
C3	0.1848 (4)	0.80391 (12)	0.70162 (6)	0.0443 (4)	
C4	0.3144 (5)	0.83206 (12)	0.65149 (7)	0.0502 (4)	
H4A	0.3157	0.8995	0.6407	0.060*	
C5	0.4418 (4)	0.75795 (12)	0.61790 (6)	0.0474 (4)	
H5A	0.5311	0.7757	0.5841	0.057*	
C6	0.4388 (4)	0.65682 (11)	0.63385 (6)	0.0397 (4)	
C7	0.5886 (4)	0.58109 (12)	0.59466 (6)	0.0437 (4)	
C8	0.5671 (4)	0.47246 (12)	0.60636 (7)	0.0465 (4)	
H8A	0.4609	0.4501	0.6380	0.056*	
C9	0.6995 (4)	0.40594 (13)	0.57171 (6)	0.0461 (4)	
H9A	0.8020	0.4332	0.5409	0.055*	
C10	0.7052 (4)	0.29630 (12)	0.57557 (6)	0.0441 (4)	
C11	0.8374 (4)	0.24150 (13)	0.53176 (7)	0.0498 (4)	
H11A	0.9211	0.2760	0.5015	0.060*	
C12	0.8463 (5)	0.13763 (14)	0.53240 (8)	0.0573 (5)	
H12A	0.9314	0.1015	0.5029	0.069*	
C13	0.7264 (5)	0.08894 (13)	0.57778 (7)	0.0571 (5)	
C14	0.5966 (5)	0.13871 (13)	0.62234 (7)	0.0571 (5)	
H14A	0.5188	0.1032	0.6526	0.069*	
C15	0.5851 (4)	0.24251 (13)	0.62076 (7)	0.0504 (4)	
H15A	0.4958	0.2776	0.6503	0.061*	

### Atomic displacement parameters (Å^2^)

**Table:**

	*U*^11^	*U*^22^	*U*^33^	*U*^12^	*U*^13^	*U*^23^
F1	0.1376 (12)	0.0406 (6)	0.0943 (9)	0.0055 (6)	0.0442 (9)	0.0029 (6)
O1	0.1010 (11)	0.0756 (10)	0.0608 (8)	0.0045 (8)	0.0352 (8)	−0.0084 (7)
O2	0.1626 (18)	0.0471 (9)	0.1018 (12)	0.0065 (9)	0.0579 (12)	−0.0095 (8)
O3	0.0962 (10)	0.0503 (7)	0.0550 (7)	−0.0027 (7)	0.0350 (7)	0.0017 (6)
N1	0.0665 (10)	0.0540 (10)	0.0534 (9)	0.0024 (7)	0.0107 (7)	−0.0086 (7)
C1	0.0552 (10)	0.0401 (9)	0.0430 (8)	−0.0028 (7)	0.0065 (7)	0.0049 (6)
C2	0.0546 (9)	0.0502 (10)	0.0395 (8)	−0.0036 (7)	0.0103 (7)	0.0030 (7)
C3	0.0465 (9)	0.0449 (9)	0.0417 (8)	−0.0009 (7)	0.0050 (7)	−0.0049 (7)
C4	0.0622 (10)	0.0374 (8)	0.0515 (9)	−0.0012 (7)	0.0107 (8)	0.0028 (7)
C5	0.0580 (10)	0.0443 (9)	0.0406 (8)	−0.0030 (7)	0.0110 (7)	0.0055 (7)
C6	0.0409 (8)	0.0398 (8)	0.0386 (8)	−0.0025 (6)	0.0027 (6)	0.0008 (6)
C7	0.0469 (9)	0.0449 (9)	0.0397 (8)	−0.0027 (7)	0.0056 (6)	0.0004 (6)
C8	0.0502 (9)	0.0438 (9)	0.0459 (8)	−0.0013 (7)	0.0098 (7)	0.0018 (7)
C9	0.0475 (9)	0.0464 (9)	0.0447 (8)	−0.0016 (7)	0.0061 (7)	0.0018 (7)
C10	0.0424 (8)	0.0450 (9)	0.0450 (8)	0.0008 (7)	0.0056 (7)	−0.0011 (7)
C11	0.0562 (10)	0.0493 (10)	0.0449 (9)	0.0016 (7)	0.0155 (7)	0.0020 (7)
C12	0.0689 (11)	0.0505 (10)	0.0538 (10)	0.0072 (8)	0.0206 (9)	−0.0059 (8)
C13	0.0689 (12)	0.0398 (9)	0.0634 (11)	0.0028 (8)	0.0151 (9)	0.0014 (8)
C14	0.0711 (12)	0.0503 (10)	0.0512 (10)	−0.0003 (8)	0.0194 (8)	0.0073 (8)
C15	0.0586 (10)	0.0494 (10)	0.0443 (9)	0.0032 (7)	0.0147 (7)	−0.0024 (7)

### Geometric parameters (Å, º)

**Table:**

F1—C13	1.362 (2)	C7—C8	1.468 (2)
O1—N1	1.2147 (19)	C8—C9	1.331 (2)
O2—N1	1.221 (2)	C8—H8A	0.9300
O3—C7	1.2204 (19)	C9—C10	1.454 (2)
N1—C3	1.473 (2)	C9—H9A	0.9300
C1—C2	1.385 (2)	C10—C11	1.397 (2)
C1—C6	1.393 (2)	C10—C15	1.398 (2)
C1—H1A	0.9300	C11—C12	1.375 (2)
C2—C3	1.374 (2)	C11—H11A	0.9300
C2—H2A	0.9300	C12—C13	1.369 (3)
C3—C4	1.381 (2)	C12—H12A	0.9300
C4—C5	1.377 (2)	C13—C14	1.374 (2)
C4—H4A	0.9300	C14—C15	1.375 (2)
C5—C6	1.393 (2)	C14—H14A	0.9300
C5—H5A	0.9300	C15—H15A	0.9300
C6—C7	1.510 (2)		
			
O1—N1—O2	122.95 (16)	C9—C8—C7	120.02 (14)
O1—N1—C3	118.80 (16)	C9—C8—H8A	120.0
O2—N1—C3	118.24 (15)	C7—C8—H8A	120.0
C2—C1—C6	120.53 (14)	C8—C9—C10	128.67 (15)
C2—C1—H1A	119.7	C8—C9—H9A	115.7
C6—C1—H1A	119.7	C10—C9—H9A	115.7
C3—C2—C1	118.60 (14)	C11—C10—C15	118.08 (15)
C3—C2—H2A	120.7	C11—C10—C9	118.33 (14)
C1—C2—H2A	120.7	C15—C10—C9	123.59 (14)
C2—C3—C4	122.43 (15)	C12—C11—C10	121.34 (15)
C2—C3—N1	119.47 (14)	C12—C11—H11A	119.3
C4—C3—N1	118.09 (15)	C10—C11—H11A	119.3
C5—C4—C3	118.43 (15)	C13—C12—C11	118.06 (16)
C5—C4—H4A	120.8	C13—C12—H12A	121.0
C3—C4—H4A	120.8	C11—C12—H12A	121.0
C4—C5—C6	120.95 (14)	F1—C13—C12	118.38 (16)
C4—C5—H5A	119.5	F1—C13—C14	118.37 (16)
C6—C5—H5A	119.5	C12—C13—C14	123.25 (17)
C5—C6—C1	119.06 (14)	C13—C14—C15	118.00 (15)
C5—C6—C7	117.17 (13)	C13—C14—H14A	121.0
C1—C6—C7	123.78 (14)	C15—C14—H14A	121.0
O3—C7—C8	121.43 (15)	C14—C15—C10	121.26 (15)
O3—C7—C6	118.36 (14)	C14—C15—H15A	119.4
C8—C7—C6	120.20 (13)	C10—C15—H15A	119.4
			
C6—C1—C2—C3	0.0 (3)	C1—C6—C7—C8	6.1 (2)
C1—C2—C3—C4	0.4 (3)	O3—C7—C8—C9	−0.8 (3)
C1—C2—C3—N1	179.54 (15)	C6—C7—C8—C9	−179.99 (15)
O1—N1—C3—C2	−7.6 (2)	C7—C8—C9—C10	−179.96 (15)
O2—N1—C3—C2	172.67 (18)	C8—C9—C10—C11	175.31 (17)
O1—N1—C3—C4	171.54 (17)	C8—C9—C10—C15	−4.6 (3)
O2—N1—C3—C4	−8.2 (3)	C15—C10—C11—C12	0.8 (3)
C2—C3—C4—C5	−0.6 (3)	C9—C10—C11—C12	−179.15 (16)
N1—C3—C4—C5	−179.74 (15)	C10—C11—C12—C13	−1.0 (3)
C3—C4—C5—C6	0.3 (3)	C11—C12—C13—F1	−179.66 (17)
C4—C5—C6—C1	0.1 (3)	C11—C12—C13—C14	0.4 (3)
C4—C5—C6—C7	−179.16 (15)	F1—C13—C14—C15	−179.50 (17)
C2—C1—C6—C5	−0.3 (2)	C12—C13—C14—C15	0.5 (3)
C2—C1—C6—C7	178.91 (15)	C13—C14—C15—C10	−0.7 (3)
C5—C6—C7—O3	6.1 (2)	C11—C10—C15—C14	0.1 (3)
C1—C6—C7—O3	−173.10 (16)	C9—C10—C15—C14	−179.98 (16)
C5—C6—C7—C8	−174.65 (15)		

### Hydrogen-bond geometry (Å, º)

**Table:**

*D*—H···*A*	*D*—H	H···*A*	*D*···*A*	*D*—H···*A*
C4—H4*A*···F1^i^	0.93	2.53	3.183 (2)	128
C11—H11*A*···O3^ii^	0.93	2.43	3.329 (2)	161
C15—H15*A*···O1^iii^	0.93	2.58	3.489 (2)	166

Symmetry codes: (i) *x*, *y*+1, *z*; (ii) −*x*+2, −*y*+1, −*z*+1; (iii) −*x*, *y*−1/2, −*z*+3/2.
